# Capillary Dysfunction: Its Detection and Causative Role in Dementias and Stroke

**DOI:** 10.1007/s11910-015-0557-x

**Published:** 2015-05-09

**Authors:** Leif Østergaard, Sune Nørhøj Jespersen, Thorbjørn Engedahl, Eugenio Gutiérrez Jiménez, Mahmoud Ashkanian, Mikkel Bo Hansen, Simon Eskildsen, Kim Mouridsen

**Affiliations:** Center of Functionally Integrative Neuroscience and MINDLab, Institute of Clinical Medicine, Aarhus University, Aarhus, Denmark; Department of Neuroradiology, Aarhus University Hospital, Building 10G, 5th Floor, Nørrebrogade 44, 8000 Aarhus, Denmark; Department of Physics and Astronomy, Aarhus University, Aarhus, Denmark

**Keywords:** Capillary dysfunction, Cardiovascular risk factors, Stroke, Reperfusion injury, Vascular dementia, Alzheimer’s disease, Perfusion imaging

## Abstract

In acute ischemic stroke, critical hypoperfusion is a frequent cause of hypoxic tissue injury: As cerebral blood flow (CBF) falls below the ischemic threshold of 20 mL/100 mL/min, neurological symptoms develop and hypoxic tissue injury evolves within minutes or hours unless the oxygen supply is restored. But is ischemia the *only* hemodynamic source of hypoxic tissue injury?

Reanalyses of the equations we traditionally use to describe the relation between CBF and tissue oxygenation suggest that capillary flow patterns are crucial for the efficient *extraction* of oxygen: without close capillary flow control, “functional shunts” tend to form and some of the blood’s oxygen content in effect becomes inaccessible to tissue.

This phenomenon raises several questions: Are there in fact two hemodynamic causes of tissue hypoxia: Limited blood supply (ischemia) and limited oxygen extraction due to *capillary dysfunction*? If so, how do we distinguish the two, experimentally and in patients? Do flow-metabolism coupling mechanisms adjust CBF to optimize tissue oxygenation when capillary dysfunction impairs oxygen extraction downstream?

Cardiovascular risk factors such as age, hypertension, diabetes, hypercholesterolemia, and smoking increase the risk of both stroke and dementia. The capillary dysfunction phenomenon therefore forces us to consider whether changes in capillary morphology or blood rheology may play a role in the etiology of some stroke subtypes and in Alzheimer’s disease.

Here, we discuss whether certain disease characteristics suggest capillary dysfunction rather than primary flow-limiting vascular pathology and how capillary dysfunction may be imaged and managed.

## Introduction

Traditionally, physiology textbooks use the extraction properties of single capillaries to arrive at the relation between tissue blood flow and tissue oxygenation [1]. Broadly stated, this *flow*-*oxygenation relation* assumes that (1) Brain parenchyma receives sufficient oxygen to support its functions if cerebral blood flow (CBF) remains above a certain threshold, extracting oxygen according to its needs. (2) Brain parenchyma receives more oxygen if the CBF increases. These properties form the rationale when we examine patients to identify hemodynamic causes of their symptoms: In patients suspected of stenoocclusive disease, we use ultrasonic and angiographic techniques to examine whether the patency of vessels in the head and neck may limit the brain’s blood supply, and CBF-sensitive methods to test whether vasodilators augment CBF, indicating that the vascular system holds a certain reserve capacity to support brain activity [2]. At a more conceptual level, we test the flow-oxygenation relation to identify and define “vascular” diseases. In Alzheimer’s disease (AD), for example, CBF decreases in the years before symptoms develop [3, 4], but not to a critical level. AD is therefore classified as a “neurodegenerative” disorder and ascribed to the presence of neurotoxic amyloid and tau species in brain parenchyma [5]. However, it remains unexplained why AD is more prevalent in patients with cardiovascular risk factors [6, 7].

In this review, we explain why the flow-oxygenation relation in textbooks is unlikely to be valid in brain tissue [8, 9••], and why the way we apply it in medical research and clinical practice may no longer hold if capillary flow patterns become disturbed [9••, 10]. We then discuss how this finding may affect our approach to diagnose and study stroke and dementias, and our understanding of the etiology of these diseases.

## Capillary Flow Patterns and Tissue Oxygenation

Textbook physiology assumes that all tissue capillaries have identical extraction properties [1]. In fact, capillary flow velocities are highly heterogeneous in a normal, resting brain [11–13]. This phenomenon can result in “functional shunting” of oxygen, glucose, and other diffusible substances through brain parenchyma: that is, their extraction becomes less efficient as capillary flows become more heterogeneous [9••, 14]. In fact, capillary flow velocities become more homogenous when brain activation causes focal hyperemia [11, 13, 15, 16], and this facilitates more efficient oxygen extraction [9••]. This homogenization is an intrinsic property of microvascular networks [17•] and seemingly ensures efficient oxygen extraction across a range of blood flows and metabolic needs in both the heart and brain [8, 9••, 18•].

### What Determines Capillary Flow Patterns?

Different cell types, structures, and biophysical quantities affect capillary blood flow patterns. *Endothelial cells* are electrically and metabolically coupled to each other and to nearby vascular smooth muscle cells (VSMC) via gap junctions composed of connexins [19]. This signaling pathway is rapid and bidirectional, and seemingly coordinates the distribution of flows across the microvascular bed [20]. Importantly, disruption of this pathway causes extreme degrees of capillary shunting through the shortest arteriolo-venular pathways [21]. *Pericytes* are contractile cells embedded in the *basement membrane* that surrounds the capillary endothelium [22]. Pericytes dilate during functional activation and control changes in both CBF and in capillary flow patterns [23••]. Pericytes and endothelial cells jointly form and maintain the basement membrane [22]. The dimensions of white blood cells and erythrocytes exceed the average capillary diameter. Local variations in basement membrane thickness, and in the shape of the cylinder it forms, are therefore likely to affect the passage of blood cells through individual capillaries. Meanwhile, the capillary endothelium’s luminal surface is covered by a 0.5-μm-thick *glycocalyx* [24]. This carbohydrate-rich matrix affects the passage of blood cells through the capillary bed [25], and glycocalyx damage is known to disrupt capillary flow regulation [26]. *Blood rheology* is also an important determinant of capillary flow patterns: Experimental studies have shown that the size, deformability, number, and endothelial adhesion of blood cells affect capillary flow patterns: in infections and low-grade vascular inflammation, blood is increasingly redirected to thoroughfare channels which act as functional shunts [27].

## Capillary Dysfunction

Capillary dysfunction denotes the failure of capillary flows to homogenize during episodes of increased blood flow [28•]. Table 1 lists a number of factors that would be expected to affect capillary flow patterns in relation to stroke, dementias, and their risk factors (see also [28•, 49•]). The critical feature of capillary dysfunction is not only the limited flow through “narrow” or “constricted” capillaries. Such capillary “micro-ischemia” is to some extent compensated by higher extraction of oxygen from the blood traveling at low velocities through the microvasculature though widespread capillary narrowing may cause ischemia or “no reflow” [50•]. For erythrocytes that pass through capillaries at *high* velocity, however, oxygen extraction efficacy can become negligible. In cases of deficient capillary control, blood flow increases induced by brain activation or hypercapnia tend to increase “functional shunting” through the capillary bed along such paths. In fact, our analysis shows that uncontrolled hyperemia can be accompanied by a paradoxical *reduction* in tissue oxygenation [9••, 18•], a phenomenon akin to the so-called luxury perfusion syndrome sometimes observed after reperfusion of ischemic tissue [51].

**Table 1:** Putative sources of capillary dysfunction in cardiovascular risk factors

Risk factor	Changes in capillary morphology or blood rheology
Aging	Human brain: Pericyte loss. Variable capillary diameters, increased capillary tortuosity, twisting, and looping. Thickened basement membranes with inclusions. Pericapillary fibrosis [29, 30]
Hypertension	Animal brain: Pericyte degeneration, swelling of endothelium and surrounding astrocyte end feet. Thickened basement membranes [31, 32] In vitro: Angiotensin II, endothelin-1 constrict retinal pericytes [33, 34]
Diabetes	Human: Thickened basement membranes [35, 36]. Hyperviscosity and reduced erythrocyte deformability in proportion to microvascular complications [37] Animal models: Pericyte loss and thickening of capillary basement membrane [31, 38, 39]. Glycocalyx degradation by oxidative stress, oxidized lipoproteins, and hyperglycemia [40–42] In vitro: Hyperglycemia-induced oxidative stress in the pericytic mitochondria causes pericyte apoptosis [43]
Alzheimer’s disease APOE ε4 genotype	Human brain: Pericyte degeneration, pericapillary fibrosis [44] In vitro: Cultured human pericytes undergo degeneration when exposed to certain subtypes of Aβ [45]. Pericytes express Aβ receptors involved in amyloid internalization and pericyte death [46]
Nicotine use (including smoking)	Nicotine upregulates the expression of adhesion molecules in the capillary endothelium [47] and increases leukocyte rolling [48]

### Quantifying Oxygen Extraction Efficacy from Capillary Flow Patterns

To quantify the effects of capillary dysfunction on tissue oxygenation, the distribution of capillary flows or capillary transit times through the capillary bed must be known [9••, 17•, 18•, 52]. The mean values of these distributions are “macroscopic” parameters, namely CBF and mean transit time (MTT), respectively. It is the microscopic variation of flow velocities or capillary transit times relative to these mean values that may cause capillary dysfunction. Below, we describe the phenomenon in terms of capillary transit time heterogeneity (CTH), but note that the phenomenon can be described in terms of flow heterogeneity as well [10, 17•, 52]. MTT is proportional to the capillary blood volume (CBV) and related to CBF through the central volume theorem: MTT = CBV/CBF [53]. When calculating the oxygen extraction efficacy for different transit time distributions, we use realistic distributions of capillary transit times that can be characterized by two parameters [9••, 17•, 18•]. This permits us to describe blood flow in terms of MTT while the standard deviation of capillary transit times serves as a measure of CTH.

In idealized capillary networks, CTH and MTT co-vary so that their ratio, the transit time coefficient of variation (CoV), tends to remain constant [17•]. When considering whether certain hemodynamic conditions reflect capillary dysfunction, both CTH and MTT should therefore be taken into account. Our model suggests that biophysically, the degree of “functional shunting” becomes critical as the standard deviation of capillary transit times (CTH) exceeds the mean (MTT) [9••, 18•]. The magnitude of CoV may therefore serve as one way to characterize capillary dysfunction.

### Flow-Metabolism Coupling and Capillary Dysfunction

Neurovascular coupling mechanisms adjust CBF according to the metabolic needs of tissue [54]. As extraction efficacy worsens with increasing capillary dysfunction, these mechanisms would therefore be expected to adjust both resting CBF and CBF responses accordingly. If capillary flow patterns are only slightly disturbed, such that their homogenization is only slightly affected during hyperemia, we expect that the accompanying reduction in oxygen extraction efficacy can be overcome by increasing CBF responses slightly—keeping in mind that oxygen delivery is the product of CBF and the oxygen extraction fraction (OEF). Unlike conditions that limit *arteriolar* diameter and thereby CBF, mild *capillary* dysfunction is therefore predicted to elicit *hyperemia* in preclinical disease. Early resting hyperemia and exaggerated CBF responses to functional activation are indeed observed in stroke and dementia risk factors: In streptozotocin-induced diabetes in rats, CBF is elevated compared to control animals early after induction of the disease [55–57]. In early-stage hypertension, CBF and BOLD responses^1^ to hypercapnia are elevated compared to control animals [58]. In asymptomatic carriers of the APOE ε4 AD risk gene aged 19–28, both resting and activity-related CBF levels are elevated [59, 60]. Similarly, BOLD signal changes during memory encoding tasks are elevated in asymptomatic APOE ε4 carries [61–63].

As capillary dysfunction becomes more severe, our analysis shows that the metabolic needs of brain tissue can only be met by limiting flow responses and, ultimately, resting CBF. In this way, the functional shunting of oxygenated blood through capillaries with fast flow velocities is reduced and more oxygen is thereby accessible for tissue metabolism. Interestingly, this transition, from hyper- to hypoperfusion, was in fact observed in a long-term follow-up study of asymptomatic APOE ε4 carriers and controls [64].

We speculate that intrinsic mechanisms attenuate CBF to limit “functional shunting” and improve net oxygen extraction in capillary dysfunction. In fact, our analyses show that if CTH increases to the highest value for which the metabolic needs of resting brain tissue can be met, CTH^max^, then the corresponding, “optimal” CBF value is close to the “classical’ ischemic threshold of 20 mL/100 mL/min [49•]. The CBF threshold at which stroke-like symptoms occur in other words applies to flow-limiting conditions as well as capillary dysfunction. The crucial difference lies in the prediction that the low CBF in capillary dysfunction is the result of flow-metabolism coupling rather than a thromboembolic event or other flow-limiting conditions. Also, attempts to “normalize” CBF in severe capillary dysfunction are, paradoxically, predicted to result in hypoxic injury due to excessive functional shunting.

## Capillary Dysfunction Signatures

The tendency of CTH to increase passively with low CBF on one hand, and the claim that flow-metabolism coupling dictates CBF to be lower in severe capillary dysfunction on the other, makes it difficult to determine whether capillary dysfunction or a flow-limiting condition is the primary culprit in a specific disease condition: Abnormal CBF and CTH values would be expected in either case. To make matters worse, the attenuation of CBF responses in hypertension and cerebral amyloidosis seemingly involves nitric oxide (NO) depletion through reactions with reactive oxygen species (ROS) [65, 66], which leads to VSMC remodeling and vascular narrowing over time [67]. The characteristic cerebral small-vessel disease (SVD) changes in arterioles and small arteries observed in the risk factors listed in Table 1 may therefore be secondary to the capillary changes downstream.

Above, we discussed how *presymptomatic hyperemia* is consistent with early capillary dysfunction, but difficult to reconcile with an evolving, flow-limiting condition. Similarly, neurological deterioration or hypoxic tissue injury at *high* CBF is inconsistent with ischemia but could be related to excessive (uncompensated) functional shunting. For example, obstructive sleep apnea (OSA) is associated with periods of severe nocturnal hypercapnia and hypoxemia, both of which cause dramatic increases in CBF in the normal brain. In addition, reductions in oxygen saturation cause a proportionate reduction in tissue oxygenation, increasing the risk of neurological symptoms or tissue injury if net oxygen extraction is already impaired. We propose that the risk, which hyperemia poses to patients who suffer from capillary dysfunction, partly explains the obervation that continuous positive airway pressure (CPAP) treatment of OSA patients reduces their incidence of both fatal and nonfatal strokes [68]. Fabry’s disease and mitochondrial encephalopathy with lactic acidosis and stroke-like episodes (MELAS) are both cerebral small-vessel diseases with a high risk of stroke and cognitive decline [69]. The tissue injury mechanism has been studied in Fabry’s disease, where *hyperemia* seemingly antedates the development of white matter lesions [70]. This has also been observed in MELAS [71].

Another characteristic of capillary dysfunction is that increases in blood viscosity may cause neurological deterioration or tissue injury in patients with pre-existing, severe capillary dysfunction, that is, tissue regions with CTH close to CTH^max^. Infections increase blood viscosity and would therefore be expected to carry a high risk of neurological deterioration and hypoxic tissue injury in patients severely affected by the risk factors listed in Table 1, particularly if infections also impair arterial oxygen saturation. We speculate that the reduction in stroke deaths observed when influenza vaccinations are offered to the elderly to avoid secondary infections [72–74] may reflect the prevention of further capillary dysfunction, and hence stroke risk. Cardiovascular risk factors also affect the microcirculation of organs outside the brain, including the heart and kidneys. We speculate that acute exacerbation of severe systemic capillary dysfunction may contribute to the simultaneous occurrence of myocardial damage in acute stroke patients and to the poor prognosis of these patients [75, 76].

## Measuring CTH and MTT

In patients with ischemic stroke, perfusion-weighted imaging (PWI) is occasionally performed in order to identify focal cerebral hypoperfusion. PWI utilizes dynamic computerized tomography (CT) or magnetic resonance (MR) images acquired after intravenous bolus injection of intravascular contrast agents that distribute in plasma [77]. Using the concentration-time curve (CTC) derived from arterial voxels identified within the imaging slices, tissue perfusion can be characterized by adjusting each imaging voxel’s CTC for the shape of the arterial CTC in individual patients—so-called deconvolution [78]. In recent years, the mean vascular transit time derived from this deconvolved CTC, as well as its delay (referred to as *T*_max_) [79, 80], have been proposed as biomarkers when deciding on intravenous thrombolysis and endovascular therapy.

CTH and MTT are variables of the transit time distributions which we use when modeling oxygen extraction in tissue. When convolved with the arterial CTC we measure with PWI [81], these distributions *also* predict the retention of tracer in tissue [82]. We can therefore determine CTH and MTT in each voxel based on our PWI data [83••] and then create maps of the corresponding oxygen extraction efficacy, OEF^max^, based on our oxygen extraction models [9••, 17•, 18•]. The method has certain limitations: First, we cannot measure oxygen metabolism by PWI, so we measure OEF^max^—not “true” oxygen extraction fraction (OEF). Second, iodinated CT contrast agents, and the Gadolinium chelates used for diagnostic magnetic resonance imaging (MRI), all distribute in plasma, whereas oxygen transport is determined by the hemodynamics of erythrocytes. In a normal brain, plasma and erythrocyte flows show comparable heterogeneities [84], but in disease, hindered erythrocyte passage through some capillaries may cause the distribution of plasma tracers to deviate from that of blood cells. Third, PWI image voxels typically contain several cubic millimeters of tissue, and their CTCs therefore reflect the retention of tracer in small arteries and veins, as well as tens of millions of capillary paths. The approach therefore has to assume that the underlying hemodynamics are dominated by uniformly affected capillary flow changes.

**Fig. 1:**
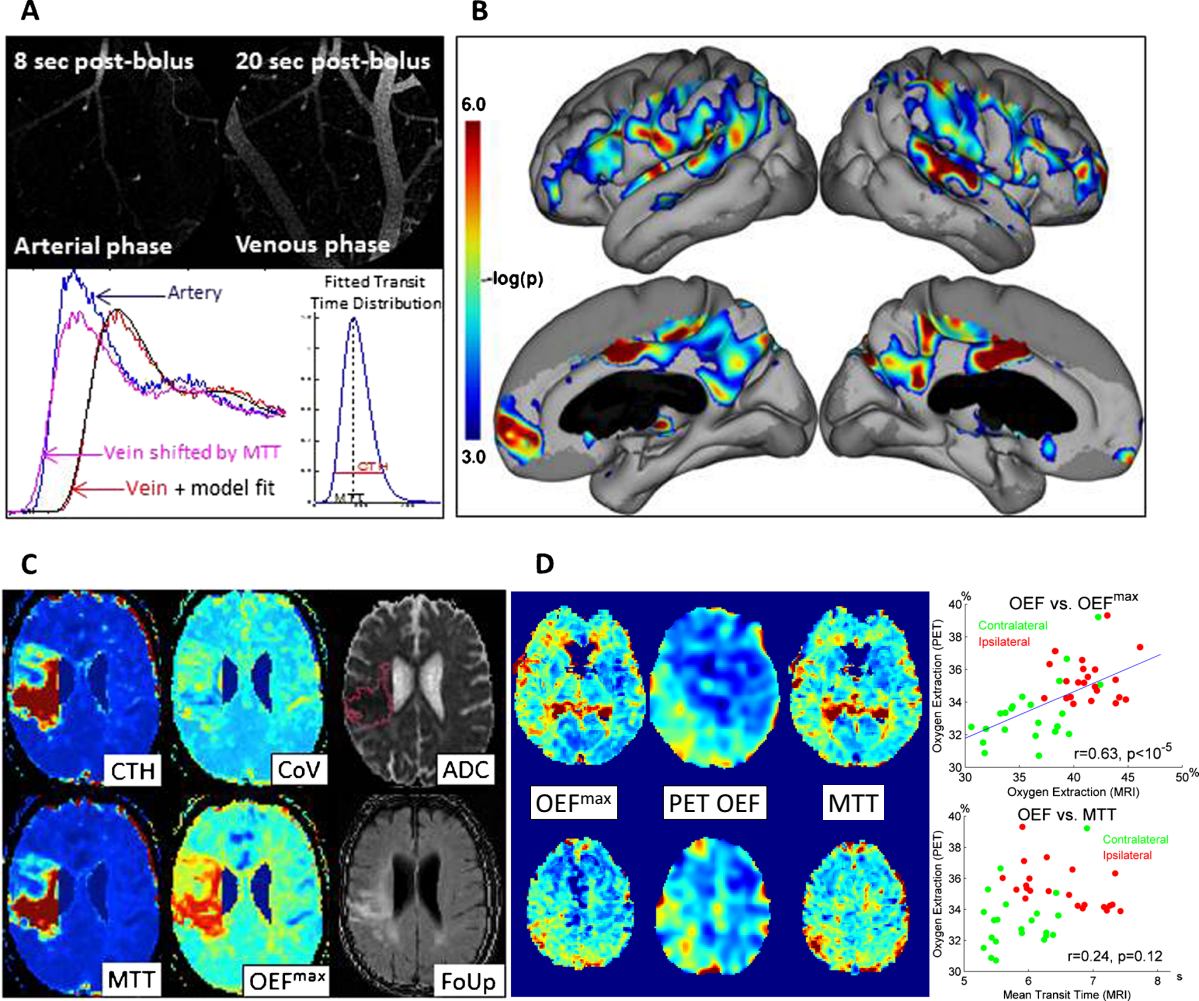
Panel **a** illustrates how MTT and CTH can be determined by two-photon microscopy, tracking the passage of a fluorescein isothiocyanate (FITC) bolus as it passes through the microvasculature of a mouse brain. The *top panels* show images obtained through a cranial window 8 and 20 s after injection. Arteries and veins can be identified based on the arrival of the fluorescent dye and their concentration-time curves (CTC) measured over time (*lower left panel*). The parameters of the transit time distribution (*lower right*) are then fitted so that the venous outflow curve is accurately predicted (*lower left panel*). The delay between arterioles and venules defines MTT, and CTH is defined as the standard deviation of the transit time distribution (*lower right panel*). Previously unpublished data. Panel **b** shows the probability that CoV correlated with mini-mental state examination (*MMSE*) scores in 16 patients with clinically suspected possible or probable Alzheimer’s disease (*AD*). Note the significant, negative correlations between MMSE and cortical CoV. AD was verified by ICD-10, DSM-IV, and NINCDS-ADRDA. The age of the patients was 70.4 ± 6.3 years and their MMSE 24.8 ± 2.7. Note the strong temporoparietal, cingulate, and precuneus involvement. In AD, these regions typically reveal abnormally low fluorodeoxyglucose uptake and cortical thinning. Data were acquired after informed consent in a project approved by the regional Ethics Committee. Previously unpublished data. Panel **c** shows maps of acute MTT, CTH, CoV, OEF^max^, and apparent diffusion coefficient (ADC) in a 74-year-old male with a distal occlusion of the middle cerebral artery, imaged 4 h and 14 min after symptom onset. His NIHSS score was 14. Twenty days later, follow-up (FoUp) FLAIR images were acquired to assess the extent of tissue damage. The extent of hypoperfusion is visualized as areas of prolonged MTT (*green*, *yellow*, and *orange colors* indicate higher values), while disturbed capillary flow patterns can be identified as elevated CTH values. Note that white matter hyperintensities, indicative of cerebral small-vessel disease (SVD) are present in this patient. These can be recognized as confluent hyperintensities in the ADC image and bright lesions in FLAIR images. Reproduced from [83••] with permission from the publisher. Panel **d** shows PET and MR data from two patients with occlusion of their right carotid artery. Both had experienced short episodes of left-sided hemiparesis and right-sided amaurosis fugax (blindness), but experienced no neurological deficits at the time of the study. Occlusion of their carotid arteries was diagnosed by ultrasonic examination, and competing cerebral pathologies were excluded by earlier MRI. The PET protocol, experimental procedures, and PET data have previously been published in [86]. The PWI data presented here were obtained after informed consent as part of the original study protocol which was approved by the local ethics committee. The PET images were manually registered (MNI Register, McConnell Brain Imaging Centre of the Montreal Neurological Institute, McGill University) to patient MRIs using an affine transformation. Then, PET OEF maps were generated for 11 slices, corresponding to the location of 11 midbrain PWI slices in which prolonged MTT could easily be observed. The *three panels on the left* compares MTT and OEF^max^ images, obtained by MRI, to OEF maps obtained by PET, at two slice locations in one of the patients. In the *two plots to the right*, the ability of MTT and OEF^max^ to predict “true” OEF is compared for the hemispheres ipsi- and contralateral to the stenosis (*red and green dots*) in 11 slices each of the two patients. The higher OEF in the affected side has traditionally been associated with severe hypoperfusion caused by carotid stenosis. See text. Previously unpublished data

In preclinical research, two-photon microscopy (TPM) can be combined with fluorescent dye injections to obtain transit time characteristics across individual microvessels in animal models [13, 85]. By comparing CTCs from arterial and venous microvessels, CTH and MTT for their connecting microvascular bed can be derived by analyses analogous to those applied to PWI data (see Fig. 1a). Meanwhile, TPM can be combined with oxygen-sensitive dyes to provide estimates of oxygen tension in tissue and blood [87] and thereby comprehensive characterization of oxygen transport in resting and activated brain tissue [88]. Recently, optical coherence tomography methods have also been refined to measure erythrocyte velocities in brain tissue [89]. This approach overcomes the problem of relying on plasma dynamics to characterize cerebral hemodynamics.

## The Putative Roles of Capillary Dysfunction in Stroke and Dementias

It seems clear that capillary flow heterogeneity can act to lower the availability of oxygen to levels below the metabolic needs of brain tissue—and thereby be the cause of compromised neuronal function and, ultimately, neuronal loss. But what are the pathways that lead from severe capillary dysfunction to radically different disease phenotypes such as AD, vascular dementia, and acute stroke, if any?

### Capillary Dysfunction in Alzheimer’s Disease

Most AD risk factors are associated with changes in capillary morphology or function (see Table 1 and ref. [28•]). The degree of capillary dysfunction is the cumulative effect across all risk factors (including age) on capillary morphology and function. We speculate that the resulting, gradual increase in CTH over time may contribute to AD pathology by causing a gradual reduction in tissue oxygen tension. Hypoxia, in turn, activates inflammatory pathways [90] and directly stimulates AD-like pathology by increasing the formation of neurofibrillary tau tangles and amyloid-β (Aβ) plaques as well as by reducing the clearance of Aβ across the blood-brain barrier (BBB) [91, 92]. Figure 1b shows cortical areas in which CoV, a measure of capillary flow heterogeneity, correlates with the mini-mental state examination (MMSE) scores of 16 patients with clinically suspected possible or probable AD. We examined CoV in order to reduce the influence of low CBF (high MTT) on microvascular hemodynamics and noted an inverse correlation between MMSE and CoV in the temporoparietal cortex, the cingulate, and the precuneus. AD patients typically show reduced glucose metabolism, as measured by fluorodeoxyglucose PET, and cortical thinning, measured with volumetric MRI, in these regions. Our preliminary results therefore suggest an association between capillary dysfunction and AD and that abnormal microvascular retention of tracer in these areas cannot be explained by low CBF alone or by cortical partial volume effects.

### Cerebral Small-Vessel Disease and Vascular Dementia

Unlike Alzheimer’s disease, vascular dementia and SVD are characterized by focal lesions in the form of small subcortical infarcts, lacunes, and white matter hyperintensities [93]. Peroxynitrite, the main reaction product of NO and ROS, inactivates tissue plasminogen activator (tPA) [94]. The oxidative stress and NO depletion, which seemingly coincide with flow suppression and hypoxia in capillary dysfunction, therefore creates an environment highly conducive to local microthrombosis. Low capillary tPA levels have thus been observed in diabetes stroke models [95]. Cerebral edema forms in severe hypoxia, and we speculate local capillary compression may provide a mechanism by which neuronal injury and edema can spread once established. Further studies of tissue microstructure and capillary hemodynamics in and around SVD lesions are needed to better understand the mechanisms that give rise to these lesions.

### Stroke and Reperfusion Injury

When we model acute stroke in rodents, vascular occlusion is used as a means to reduce regional CBF by 60–90 % to inflict hypoxic tissue injury. This approach does not account for, perhaps the most important property of the capillary dysfunction phenomenon, namely the gradual accumulation of microvascular changes listed in Table 1. These evolve over several decades in patients who may ultimately suffer an acute stroke. Capillary dysfunction is also predicted to markedly reduce cerebrovascular reserve capacity—not only due to large vessel disease but also by flow-metabolism coupling that suppresses vasodilation to prevent excessive functional shunting. As a result, these patients are predicted to be extremely vulnerable to infections, to small reductions in blood pressure, and perhaps even to small emboli that reduce local perfusion only slightly and temporarily. Rather than the “before” and “after” scenario of animal stroke models, we may therefore need to consider stroke as a condition that in some patients develop over many years in much the same way as AD—only to culminate in an acute manner.

Many strokes are clearly caused by thromboembolic events. In these cases, the capillary dysfunction phenomenon may become important in relation to recanalization therapy. Ischemia is known to cause pericyte constrictions that fail to reverse after reperfusion [23••, 50•, 96]. As sudden normalization of CBF without parallel normalization of CTH is predicted to cause severe “functional shunting” and tissue hypoxia, a means of restoring capillary flow patterns may be as important as restoring overall CBF. We discuss these effects in relation to pre-stroke risk factors and the poor prognosis of tissue that displays post-recanalization hyperperfusion in [49•], and the specific issues relating to delayed cerebral ischemia after hemorrhagic stroke in [97•].

Figure 1c shows CTH, MTT, and CoV maps in an acute stroke patient and the model’s prediction of OEF^max^. Hypoperfusion can be identified as areas of elevated MTT values (yellow to orange colors). The most severe hypoperfusion can be identified as the area of reduced apparent diffusion coefficient (ADC), which is thought to reflect acute ischemic injury and cytotoxic edema. The red outline indicates the infarct volume 20 days later, in this case revealing little expansion of the acute lesion. Note that an area of low CoV can be observed within the severely ischemic tissue. The origin of this “relative homogenization,” and whether it reflects the dynamics of blood or mostly plasma, remains unclear. We have previously found that areas with relative flow homogenization are highly prone to subsequent infarction [10, 98, 99]. Note the areas of elevated CoV and OEF^max^, both within and outside the severely hypoperfused tissue region. White matter hyperintensities, visible as bright areas posterior to the ventricles on acute ADC maps (suggestive of extracellular edema), and on follow-up FLAIR seem to be located in areas of elevated COV and OEF^max^, suggesting an association between capillary dysfunction and these SVD lesions.

Further studies are needed to fully understand how macrovascular (as measured by MTT) and microvascular (as measured by CTH) hemodynamics contribute to infarct progression and to ascertain whether capillary dysfunction contributes to certain lesion types, to certain clinical presentations, such as transitory ischemic attacks (TIA), or to progressive cognitive decline before the acute stroke occurs.

## Therapeutic and Diagnostic Challenges

In patients suspected of cerebrovascular disease, the patency of carotid and intracranial vessels are often examined by ultrasound or by angiographic techniques. Vascular occlusions or high-grade vascular stenoses may cause critical reductions in CBF, and in patients who reveal such vascular changes, cerebrovascular and metabolic reserve capacity is often measured to examine their severity. It is generally assumed that blood flow is “mechanically” limited by such occlusions and that the presence of macroscopic increased oxygen extraction fraction (OEF), a classical marker of misery perfusion, is a certain sign of ischemia. The capillary dysfunction is also expected to attenuate CBF and to result in elevated OEF at a microscopic level, however, and this prediction may have profound diagnostic and therapeutic implications in patients with cerebrovascular disease.

Figure 1d shows OEF maps from two slice positions in a patient with carotid stenosis. They were acquired by PET and O-15-labeled water and oxygen administration. Using our model [9••], we also estimated OEF^max^ based on maps of CTH and MTT [83••]. If the patient’s elevated OEF could be explained by restricted blood supply alone, we would expect OEF to correlate with MTT. If capillary dysfunction also affects OEF, however, we would expect the OEF^max^ model that takes CTH into account, to better explain actual OEF levels. The graphs to the right show the correlations between “true” OEF on one hand and MTT and OEF^max^, respectively, on the other. The points represent entire hemisphere values ipsilateral (red) and contralateral (green) to the stenosis in 11 slices of two carotid stenosis patients. As in the acute stroke case above, it appears that both capillary dysfunction and ischemia affect brain oxygenation and that the presence of elevated OEF cannot distinguish between the two mechanisms.

The identification of those symptomatic carotid stenosis patients who are likely to benefit from revascularization therapy based on findings of impaired cerebrovascular reserve capacity and elevated OEF has yielded mixed results [100]. We propose that quantification of concomitant capillary dysfunction could help predict the relative benefits of revascularization therapy and aggressive management of cardiovascular risk factors, of which the latter is more likely to affect the degree of capillary dysfunction.

We discuss the therapeutic and diagnostic implications of capillary dysfunction in stroke and dementia further in [28•, 49•, 97•].

## Conclusion

The effect of capillary flow patterns on oxygen extraction efficacy implies that tissue oxygenation can be reduced both as a result of limited blood supply (ischemia) and as a result of impaired oxygen extraction due to capillary dysfunction. Flow-metabolism coupling may make these phenomena difficult to distinguish, but certain disease features, such as presymptomatic hyperemia and early capillary involvement on one hand and symptoms modulated by blood rheology on the other, may suggest mild and severe capillary dysfunction, respectively. Methods to detect capillary dysfunction are currently being developed, and our preliminary findings suggest that capillary dysfunction may play a role in both cerebrovascular disease and AD. Further studies should elucidate whether capillary dysfunction represents a useful diagnostic entity or a target for prevention and therapy in these conditions.

## Footnotes

1 The blood oxygen level-dependent (BOLD) magnetic resonance imaging (MRI) signal decreases as a function of the local tissue deoxyhemoglobin concentration. Lower OEF and higher CBF both reduce deoxyhemoglobin levels. Compared to tissue where capillary flow patterns homogenize normally during a certain stimulus, BOLD response amplitudes are therefore elevated in tissue with slight capillary dysfunction during hyperemia, if metabolic needs are met by larger CBF responses.

## References

[1] Renkin EM (1985). B. W. Zweifach Award lecture. Regulation of the microcirculation. Microvasc Res.

[2] Vorstrup S, Henriksen L, Paulson OB (1984). Effect of acetazolamide on cerebral blood flow and cerebral metabolic rate for oxygen. J Clin Invest.

[3] Ruitenberg A, den Heijer T, Bakker SL (2005). Cerebral hypoperfusion and clinical onset of dementia: the Rotterdam study. Ann Neurol.

[4] Hirao K, Ohnishi T, Hirata Y (2005). The prediction of rapid conversion to Alzheimer’s disease in mild cognitive impairment using regional cerebral blood flow SPECT. Neuroimage.

[5] Hardy J, Selkoe DJ (2002). The amyloid hypothesis of Alzheimer’s disease: progress and problems on the road to therapeutics. Science.

[6] Zlokovic BV (2011). Neurovascular pathways to neurodegeneration in Alzheimer’s disease and other disorders. Nat Rev Neurosci.

[7] Iadecola C (2010). The overlap between neurodegenerative and vascular factors in the pathogenesis of dementia. Acta Neuropathol.

[8] Østergaard L, Kristiansen SB, Angleys H (2014). The role of capillary transit time heterogeneity in myocardial oxygenation and ischemic heart disease. Basic Res Cardiol.

[9.••] Jespersen SN, Østergaard L (2012). The roles of cerebral blood flow, capillary transit time heterogeneity and oxygen tension in brain oxygenation and metabolism. J Cereb Blood Flow Metab.

[10] Østergaard L, Sorensen AG, Chesler DA (2000). Combined diffusion-weighted and perfusion-weighted flow heterogeneity magnetic resonance imaging in acute stroke. Stroke.

[11] Kleinfeld D, Mitra PP, Helmchen F, Denk W (1998). Fluctuations and stimulus-induced changes in blood flow observed in individual capillaries in layers 2 through 4 of rat neocortex. Proc Natl Acad Sci U S A.

[12] Villringer A, Them A, Lindauer U, Einhaupl K, Dirnagl U (1994). Capillary perfusion of the rat brain cortex. An in vivo confocal microscopy study. Circ Res.

[13] Stefanovic B, Hutchinson E, Yakovleva V (2008). Functional reactivity of cerebral capillaries. J Cereb Blood Flow Metab.

[14] Paulson OB, Hasselbalch SG, Rostrup E, Knudsen GM, Pelligrino D (2010). Cerebral blood flow response to functional activation. J Cereb Blood Flow Metab.

[15] Schulte ML, Wood JD, Hudetz AG (2003). Cortical electrical stimulation alters erythrocyte perfusion pattern in the cerebral capillary network of the rat. Brain Res.

[16] Vogel J, Kuschinsky W (1996). Decreased heterogeneity of capillary plasma flow in the rat whisker-barrel cortex during functional hyperemia. J Cereb Blood Flow Metab.

[17.•] Rasmussen PM, Jespersen SN, Østergaard L (2015). The effects of transit time heterogeneity on brain oxygenation during rest and functional activation. J Cereb Blood Flow Metab.

[18.•] Angleys H, Østergaard L, Jespersen SN (2015). The effects of capillary transit time heterogeneity (CTH) on brain oxygenation. J Cereb Blood Flow Metab.

[19] Isakson BE, Damon DN, Day KH, Liao Y, Duling BR (2006). Connexin40 and connexin43 in mouse aortic endothelium: evidence for coordinated regulation. Am J Physiol Heart Circ Physiol.

[20] Segal SS, Duling BR (1986). Flow control among microvessels coordinated by intercellular conduction. Science.

[21] Pries AR, Hopfner M, le Noble F, Dewhirst MW, Secomb TW (2010). The shunt problem: control of functional shunting in normal and tumour vasculature. Nat Rev Cancer.

[22] Armulik A, Abramsson A, Betsholtz C (2005). Endothelial/pericyte interactions. Circ Res.

[23.••] Hall CN, Reynell C, Gesslein B (2014). Capillary pericytes regulate cerebral blood flow in health and disease. Nature.

[24] Vink H, Duling BR (1996). Identification of distinct luminal domains for macromolecules, erythrocytes, and leukocytes within mammalian capillaries. Circ Res.

[25] Secomb TW, Hsu R, Pries AR (1998). A model for red blood cell motion in glycocalyx-lined capillaries. Am J Physiol.

[26] Desjardins C, Duling BR (1990). Heparinase treatment suggests a role for the endothelial cell glycocalyx in regulation of capillary hematocrit. Am J Physiol.

[27] Mazzoni MC, Schmid-Schonbein GW (1996). Mechanisms and consequences of cell activation in the microcirculation. Cardiovasc Res.

[28.•] Østergaard L, Aamand R, Gutierrez-Jimenez E (2013). The capillary dysfunction hypothesis of Alzheimer’s disease. Neurobiol Aging.

[29] Kalaria RN (1996). Cerebral vessels in ageing and Alzheimer’s disease. Pharmacol Ther.

[30] Bell MA, Ball MJ (1981). Morphometric comparison of hippocampal microvasculature in ageing and demented people: diameters and densities. Acta Neuropathol.

[31] Junker U, Jaggi C, Bestetti G, Rossi GL (1985). Basement membrane of hypothalamus and cortex capillaries from normotensive and spontaneously hypertensive rats with streptozotocin-induced diabetes. Acta Neuropathol.

[32] Tagami M, Nara Y, Kubota A, Fujino H, Yamori Y (1990). Ultrastructural changes in cerebral pericytes and astrocytes of stroke-prone spontaneously hypertensive rats. Stroke.

[33] Schonfelder U, Hofer A, Paul M, Funk RH (1998). In situ observation of living pericytes in rat retinal capillaries. Microvasc Res.

[34] Kawamura H, Kobayashi M, Li Q (2004). Effects of angiotensin II on the pericyte-containing microvasculature of the rat retina. J Physiol.

[35] Johnson PC, Brendel K, Meezan E (1982). Thickened cerebral cortical capillary basement membranes in diabetics. Arch Pathol Lab Med.

[36] Reske-Nielsen E, Lundbæk K, Rafaelsen OJ (1965). Pathological changes in the central and peripheral nervous system of young long-term diabetics. I. Diabetic encephalopathy. Diabetologia.

[37] Barnes AJ, Locke P, Scudder PR, Dormandy TL, Dormandy JA, Slack J (1977). Is hyperviscosity a treatable component of diabetic microcirculatory disease?. Lancet.

[38] McCuskey PA, McCuskey RS (1984). In vivo and electron microscopic study of the development of cerebral diabetic microangiography. Microcirc Endothel Lymphat.

[39] Price TO, Eranki V, Banks WA, Ercal N, Shah GN (2012). Topiramate treatment protects blood-brain barrier pericytes from hyperglycemia-induced oxidative damage in diabetic mice. Endocrinology.

[40] Vink H, Constantinescu AA, Spaan JA (2000). Oxidized lipoproteins degrade the endothelial surface layer : implications for platelet-endothelial cell adhesion. Circulation.

[41] Constantinescu AA, Vink H, Spaan JA (2001). Elevated capillary tube hematocrit reflects degradation of endothelial cell glycocalyx by oxidized LDL. Am J Physiol Heart Circ Physiol.

[42] Czarnowska E, Karwatowska-Prokopczuk E (1995). Ultrastructural demonstration of endothelial glycocalyx disruption in the reperfused rat heart. Involvement of oxygen free radicals. Basic Res Cardiol.

[43] Shah GN, Price TO, Banks WA (2013). Pharmacological inhibition of mitochondrial carbonic anhydrases protects mouse cerebral pericytes from high glucose-induced oxidative stress and apoptosis. J Pharmacol Exp Ther.

[44] Farkas E, Luiten PG (2001). Cerebral microvascular pathology in aging and Alzheimer’s disease. Prog Neurobiol.

[45] Verbeek MM, de Waal RM, Schipper JJ, Van Nostrand WE (1997). Rapid degeneration of cultured human brain pericytes by amyloid beta protein. J Neurochem.

[46] Wilhelmus MMM, Otte-Höller I, van Triel JJJ (2007). Lipoprotein receptor-related protein-1 mediates amyloid-β-mediated cell death of cerebrovascular cells. Am J Pathol.

[47] Albaugh G, Bellavance E, Strande L, Heinburger S, Hewitt CW, Alexander JB (2004). Nicotine induces mononuclear leukocyte adhesion and expression of adhesion molecules, VCAM and ICAM, in endothelial cells in vitro. Ann Vasc Surg.

[48] Yong T, Zheng MQ, Linthicum DS (1997). Nicotine induces leukocyte rolling and adhesion in the cerebral microcirculation of the mouse. J Neuroimmunol.

[49.•] Østergaard L, Jespersen SN, Mouridsen K, et al. The role of the cerebral capillaries in acute ischemic stroke: the extended penumbra model. J Cereb Blood Flow Metab. 2013;33:635–48. **The authors demonstrate that a certain CTH threshold, CTH**^max^**exists for which any changes in CBF (increases or reductions) or additional capillary flow disturbances (eg. increased blood viscosity) can cause stroke-like symptoms. They propose that severe capillary dysfunction represent a condition of extreme risk of both stroke and dementia, and show that flow metabolism coupling mechanisms will cause CBF to approach the classical ischemic threshold as CTH approaches CTH**^max^. **They argue that irreversible per-ischemic capillary compression or pericyte constrictions are likely to cause severe tissue hypoxia upon reperfusion and underscore the importance of capillary reperfusion in stroke care, irrespective of whether recanalization therapy is attempted or not. They discover that the T**_max_**parameter, which is used by some for imaging-based selection for revascularization therapy, show a coincidental correlation with oxygen extraction efficacy, OEF**^max^. 10.1038/jcbfm.2013.18 PMC3652700 23443173

[50.•] Dalkara T, Arsava EM (2012). Can restoring incomplete microcirculatory reperfusion improve stroke outcome after thrombolysis?. J Cereb Blood Flow Metab.

[51] Lassen NA (1966). The luxury-perfusion syndrome and its possible relation to acute metabolic acidosis localised within the brain. Lancet.

[52] Østergaard L, Chesler DA, Weisskoff RM, Sorensen AG, Rosen BR (1999). Modeling cerebral blood flow and flow heterogeneity from magnetic resonance residue data. J Cereb Blood Flow Metab.

[53] Stewart GN (1893). Researches on the circulation time in organs and on the influences which affect it. Parts I.-III. J Physiol.

[54] Iadecola C (2004). Neurovascular regulation in the normal brain and in Alzheimer’s disease. Nat Rev Neurosci.

[55] Al-Saeedi FJ (2008). Perfusion scanning using 99mTc-HMPAO detects early cerebrovascular changes in the diabetic rat. BMC Med Phys.

[56] Simpson RE, Phillis JW, Buchannan J (1990). A comparison of cerebral blood flow during basal, hypotensive, hypoxic and hypercapnic conditions between normal and streptozotocin diabetic rats. Brain Res.

[57] Rubin MJ, Bohlen HG (1985). Cerebral vascular autoregulation of blood flow and tissue PO2 in diabetic rats. Am J Physiol.

[58] Kim T, Richard Jennings J, Kim SG (2014). Regional cerebral blood flow and arterial blood volume and their reactivity to hypercapnia in hypertensive and normotensive rats. J Cereb Blood Flow Metab.

[59] Scarmeas N, Habeck CG, Stern Y, Anderson KE (2003). APOE genotype and cerebral blood flow in healthy young individuals. JAMA.

[60] Scarmeas N, Habeck CG, Hilton J (2005). APOE related alterations in cerebral activation even at college age. J Neurol Neurosurg Psychiatry.

[61] Bookheimer SY, Strojwas MH, Cohen MS (2000). Patterns of brain activation in people at risk for Alzheimer’s disease. N Engl J Med.

[62] Fleisher AS, Podraza KM, Bangen KJ (2009). Cerebral perfusion and oxygenation differences in Alzheimer’s disease risk. Neurobiol Aging.

[63] Filippini N, MacIntosh BJ, Hough MG (2009). Distinct patterns of brain activity in young carriers of the APOE-epsilon4 allele. Proc Natl Acad Sci U S A.

[64] Thambisetty M, Beason-Held L, An Y, Kraut MA, Resnick SM (2010). APOE epsilon4 genotype and longitudinal changes in cerebral blood flow in normal aging. Arch Neurol.

[65] Girouard H, Park L, Anrather J, Zhou P, Iadecola C (2007). Cerebrovascular nitrosative stress mediates neurovascular and endothelial dysfunction induced by angiotensin II. Arterioscler Thromb Vasc Biol.

[66] Niwa K, Porter VA, Kazama K, Cornfield D, Carlson GA, Iadecola C (2001). A beta-peptides enhance vasoconstriction in cerebral circulation. Am J Physiol Heart Circ Physiol.

[67] Touyz RM, Schiffrin EL (2004). Reactive oxygen species in vascular biology: implications in hypertension. Histochem Cell Biol.

[68] Marin JM, Carrizo SJ, Vicente E, Agusti AG (2005). Long-term cardiovascular outcomes in men with obstructive sleep apnoea-hypopnoea with or without treatment with continuous positive airway pressure: an observational study. Lancet.

[69] Pantoni L (2010). Cerebral small vessel disease: from pathogenesis and clinical characteristics to therapeutic challenges. Lancet Neurol.

[70] Moore DF, Altarescu G, Barker WC, Patronas NJ, Herscovitch P, Schiffmann R (2003). White matter lesions in Fabry disease occur in ‘prior’ selectively hypometabolic and hyperperfused brain regions. Brain Res Bull.

[71] Takahashi S, Tohgi H, Yonezawa H, Obara S, Nagane Y (1998). Cerebral blood flow and oxygen metabolism before and after a stroke-like episode in patients with mitochondrial myopathy, encephalopathy, lactic acidosis and stroke-like episodes (MELAS). J Neurol Sci.

[72] Lavallee P, Perchaud V, Gautier-Bertrand M, Grabli D, Amarenco P (2002). Association between influenza vaccination and reduced risk of brain infarction. Stroke.

[73] Nichol KL, Nordin J, Mullooly J, Lask R, Fillbrandt K, Iwane M (2003). Influenza vaccination and reduction in hospitalizations for cardiac disease and stroke among the elderly. N Engl J Med.

[74] Grau AJ, Fischer B, Barth C, Ling P, Lichy C, Buggle F (2005). Influenza vaccination is associated with a reduced risk of stroke. Stroke.

[75] James P, Ellis CJ, Whitlock RM, McNeil AR, Henley J, Anderson NE (2000). Relation between troponin T concentration and mortality in patients presenting with an acute stroke: observational study. BMJ.

[76] Jensen JK, Kristensen SR, Bak S, Atar D, Hoilund-Carlsen PF, Mickley H (2007). Frequency and significance of troponin T elevation in acute ischemic stroke. Am J Cardiol.

[77] Østergaard L, Sorensen AG, Kwong KK, Weisskoff RM, Gyldensted C, Rosen BR (1996). High resolution measurement of cerebral blood flow using intravascular tracer bolus passages. Part II: experimental comparison and preliminary results. Magn Reson Med.

[78] Østergaard L, Weisskoff RM, Chesler DA, Gyldensted C, Rosen BR (1996). High resolution measurement of cerebral blood flow using intravascular tracer bolus passages. Part I: mathematical approach and statistical analysis. Magn Reson Med.

[79] Takasawa M, Jones PS, Guadagno JV (2008). How reliable is perfusion MR in acute stroke? Validation and determination of the penumbra threshold against quantitative PET. Stroke.

[80] Olivot JM, Mlynash M, Thijs VN (2009). Optimal Tmax threshold for predicting penumbral tissue in acute stroke. Stroke.

[81] Mouridsen K, Christensen S, Gyldensted L, Østergaard L (2006). Automatic selection of arterial input function using cluster analysis. Magn Reson Med.

[82] Mouridsen K, Friston K, Hjort N, Gyldensted L, Østergaard L, Kiebel S (2006). Bayesian estimation of cerebral perfusion using a physiological model of microvasculature. Neuroimage.

[83.••] Mouridsen K, Hansen MB, Østergaard L, Jespersen SN (2014). Reliable estimation of capillary transit time distributions using DSC-MRI. J Cereb Blood Flow Metab.

[84] Vogel J, Waschke KF, Kuschinsky W (1997). Flow-independent heterogeneity of brain capillary plasma perfusion after blood exchange with a Newtonian fluid. Am J Physiol.

[85] Hutchinson EB, Stefanovic B, Koretsky AP, Silva AC (2006). Spatial flow-volume dissociation of the cerebral microcirculatory response to mild hypercapnia. Neuroimage.

[86] Ashkanian M, Gjedde A, Mouridsen K (2009). Carbogen inhalation increases oxygen transport to hypoperfused brain tissue in patients with occlusive carotid artery disease: increased oxygen transport to hypoperfused brain. Brain Res.

[87] Sakadzic S, Roussakis E, Yaseen MA (2010). Two-photon high-resolution measurement of partial pressure of oxygen in cerebral vasculature and tissue. Nat Methods.

[88] Sakadzic S, Mandeville ET, Gagnon L (2014). Large arteriolar component of oxygen delivery implies a safe margin of oxygen supply to cerebral tissue. Nat Commun.

[89] Lee J, Wu W, Lesage F, Boas DA (2013). Multiple-capillary measurement of RBC speed, flux, and density with optical coherence tomography. J Cereb Blood Flow Metab.

[90] Eltzschig HK, Carmeliet P (2011). Hypoxia and inflammation. N Engl J Med.

[91] Zhang X, Le W (2010). Pathological role of hypoxia in Alzheimer’s disease. Exp Neurol.

[92] Bell RD, Deane R, Chow N (2009). SRF and myocardin regulate LRP-mediated amyloid-beta clearance in brain vascular cells. Nat Cell Biol.

[93] Wardlaw JM, Smith EE, Biessels GJ (2013). Neuroimaging standards for research into small vessel disease and its contribution to ageing and neurodegeneration. Lancet Neurol.

[94] Iadecola C, Davisson RL (2008). Hypertension and cerebrovascular dysfunction. Cell Metab.

[95] Kittaka M, Wang L, Sun N (1996). Brain capillary tissue plasminogen activator in a diabetes stroke model. Stroke.

[96] Yemisci M, Gursoy-Ozdemir Y, Vural A, Can A, Topalkara K, Dalkara T (2009). Pericyte contraction induced by oxidative-nitrative stress impairs capillary reflow despite successful opening of an occluded cerebral artery. Nat Med.

[97.•] Østergaard L, Aamand R, Karabegovic S (2013). The role of the microcirculation in delayed cerebral ischemia and chronic degenerative changes after subarachnoid hemorrhage. J Cereb Blood Flow Metab.

[98] Perkio J, Soinne L, Østergaard L (2005). Abnormal intravoxel cerebral blood flow heterogeneity in human ischemic stroke determined by dynamic susceptibility contrast magnetic resonance imaging. Stroke.

[99] Simonsen CZ, Rohl L, Vestergaard-Poulsen P, Gyldensted C, Andersen G, Østergaard L (2002). Final infarct size after acute stroke: prediction with flow heterogeneity. Radiology.

[100] Powers WJ, Clarke WR, Grubb RL (2011). Extracranial-intracranial bypass surgery for stroke prevention in hemodynamic cerebral ischemia: the Carotid Occlusion Surgery Study randomized trial. JAMA.

